# The Combined Effect of Hydrophobic Mismatch and Bilayer Local Bending on the Regulation of Mechanosensitive Ion Channels

**DOI:** 10.1371/journal.pone.0150578

**Published:** 2016-03-09

**Authors:** Omid Bavi, Manouchehr Vossoughi, Reza Naghdabadi, Yousef Jamali

**Affiliations:** 1 Institute for Nanoscience and Nanotechnology, Sharif University of Technology, Tehran, Iran; 2 Biochemical & Bioenvironmental Research Center (BBRC), Tehran, Iran; 3 Department of Mechanical Engineering, Sharif University of Technology, Tehran, Iran; 4 Department of Mathematics, Tarbiat Modares University, Tehran, Iran; 5 Computational physical Sciences Research Laboratory, School of Nano-Science, Institute for Research in Fundamental Sciences (IPM), Tehran, Iran; University of Cambridge, UNITED KINGDOM

## Abstract

The hydrophobic mismatch between the lipid bilayer and integral membrane proteins has well-defined effect on mechanosensitive (MS) ion channels. Also, membrane local bending is suggested to modulate MS channel activity. Although a number of studies have already shown the significance of each individual factor, the combined effect of these physical factors on MS channel activity have not been investigated. Here using finite element simulation, we study the combined effect of hydrophobic mismatch and local bending on the archetypal mechanosensitive channel MscL. First we show how the local curvature direction impacts on MS channel modulation. In the case of MscL, we show inward (cytoplasmic) bending can more effectively gate the channel compared to outward bending. Then we indicate that in response to a specific local curvature, MscL inserted in a bilayer with the same hydrophobic length is more expanded in the constriction pore region compared to when there is a protein-lipid hydrophobic mismatch. Interestingly in the presence of a negative mismatch (thicker lipids), MscL constriction pore is more expanded than in the presence of positive mismatch (thinner lipids) in response to an identical membrane curvature. These results were confirmed by a parametric energetic calculation provided for MscL gating. These findings have several biophysical consequences for understanding the function of MS channels in response to two major physical stimuli in mechanobiology, namely hydrophobic mismatch and local membrane curvature.

## Introduction

Studies of integral membrane proteins have demonstrated that their function is dynamically modulated by the surrounding lipid bilayer [1, 2]. Many of these membrane proteins, in particular mechanosensitive (MS) ion channels, interact with annular lipids of the bilayer via tight hydrophobic and/or electrostatic interactions [2–4]. Thus, any mechanical perturbation in the lipid bilayer has a consequent force induced conformational change in the embedded protein as well [3, 5, 6].

Previous studies of MS channels have already shown the importance of membrane tension not only in the gating of bacterial channels [7, 8] and small antibiotic peptides such as Gramicidin A [9], but also for eukaryotic channels such as TRPV4, TREK, TRAAK and PIEZO1 [10–13]. Thus, it is essential to study the effect of bilayer physical properties on the regulation of MS channels [3, 5, 14]. Two important physical factors that have a major role in the gating of MS channels are protein-lipid hydrophobic mismatch and membrane curvature.

The effect of each individual factor has previously been studied using a plethora of experimental and computational approaches (e.g. patch-clamp technique, Electron Paramagnetic Resonance spectroscopy (EPR), Fluorescence Resonance Energy Transfer (FRET) and MD simulation) [8, 15–21]. For instance, it has been shown that lipid bilayers with different acyl chain lengths have a differential impact on MscL function [17, 22, 23]. Besides MS channels, the small antibiotic peptide gramicidin A can be either activated or inactivated by tension depending on bilayer thickness [9]. In addition to tension, asymmetric incorporation of lipids (e.g. Phosphatidylinositol 4,5-bisphosphate), lysolipids (e.g. Lysophosphatidylcholine, LPC) and amphipaths (e.g. chlorpromazine, CPZ) modulate the activity of MS channels due to their asymmetrical effect on the transbilayer pressure profile [17, 18, 24–26]. Almost all of the studies in this area suggest that MS channel modulation by asymmetric insertion of amphipaths is due to their effect on local curvature of the bilayer. However, we should note that other mechanisms have been proposed as well, such as effects on line tension [27]. Other physiological processes such as cell division, endocytosis and exocytosis also involve severe degrees of local membrane bending as well as membrane thickness alteration. Thus, it is crucial to study the effect of these two membrane physical factors on MS channel activity. It is also important to note that by local curvature or local bending here we mean a radius of curvature in the range of caveolae [28] and those curvatures caused by clathrin-coated pits [29], which are normally < 100 nm. We also need to consider that only local curvature can modulate MS channel activity, whilst change in the global membrane curvature radius (i.e., > 200 nm) has a negligible effect on the pressure profile of the membrane and thus MS channel activity [5, 30, 31]. Therefore, unlike what has been suggested in previous studies [11, 32, 33], changes in the global curvature of the membrane patch, for example when positive and negative pressures have been applied to a portion of membrane in a patch-clamp set up, is not enough per se to modulate MS channel activity. The differences seen can almost always be explained by the differential generated tension in the bilayer at positive compared to negative pressures [30].

Herein we investigate two major physiological stimuli in the biophysics of the lipid bilayer, hydrophobic mismatch and local bending, and examine how an MS channel responds when both physical factors converge. To address this, we employ a representative volume element (RVE) for MscL and a continuum mechanical framework proposed in Bavi *et al*. [5] for modeling different lipid bilayers. Furthermore, we evaluate our outcomes with a theoretical formalism that calculates the energetic contribution of the hydrophobic mismatch and the bilayer local bending in the gating free energy cost at the same time.

## Materials and Methods

### Finite Element (FE) model

Due to the “multi time scale and length scale” nature of the full gating process of MscL and the variety of conditions which are considered in this study, employing atomistic simulation is expected to be computationally extensive. The idea here is to use an efficient framework of multiscale modeling to capture a conformational change on the protein scale rather than in atomistic details [34–38]. This way we avoid excessive computational costs. Moreover, using such a framework, we are able to study a number of different cases including the effect of bending in different directions for different membrane thicknesses.

**Fig 1:**
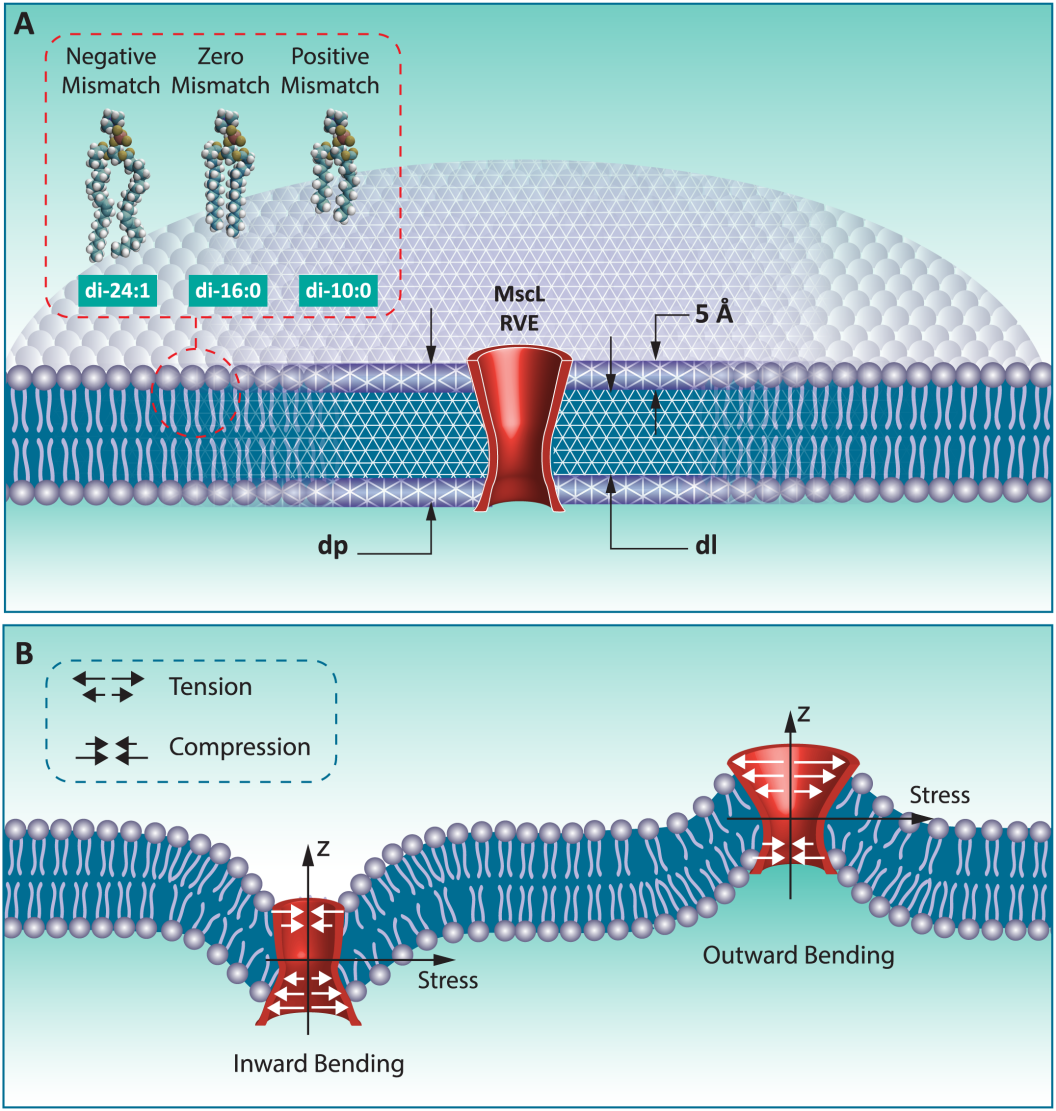
The continuum model of MscL [5] which is embedded in a continuum implicit model bilayer. (A) Lipid with three different acyl chain length (i.e. di-10:0, di-16:0 and of di-24:1) have been simulated using finite element (FE) method for positive, zero and negative mismatches between the protein and the lipid bilayer respectively. (B) The influence of inward (negative) and outward (positive) local membrane bending on the channel deformation. Depending on the direction of curvature, different parts of the channel experience tension or compression.

In our FE model, lipid bilayer has been considered as a three layer laminate, where the upper and lower sections represent the head groups and the core section represents the tails region of the lipid bilayer (Fig 1a). We were able to simplify the pressure profile of lipid bilayer with different acyl chain length in a mean field manner and assign it to each of these three sections in our model (see Bavi *et al*., 2014 [5] for the details). Moreover, the reported representative volume element (RVE) model of MscL used here was designed previously such that it is consistent with the MscL pore shape in different states, including in detergent, resting and open states. Therefore, the Young’s modulus and Poisson’s ratio of the RVE are assumed to be 40 MPa and 0.5, respectively. Our model also takes into account the hydrophobic pairwise interaction between the MscL RVE and the lipid bilayer (See Ref [5] for more details). The energies of these interactions and the ranges of the protein induced perturbations of the bilayer have previously been calculated for MscL, from molecular dynamics (MD) simulations and continuum mechanics approaches [35].

Taking all these factors into consideration enabled us to capture the phenomenological details of the MscL conformation during membrane bending for different bilayer thicknesses (i.e. different protein-lipid hydrophobic mismatch conditions) as well as different bending directions (Fig 1). However, compared to MD simulations, the atomistic details such as charge interactions, internal degrees of freedom of the protein and inter-protein interactions have obviously been in our modeling. Yet, we are confident that our framework is accurate enough to address the question raised in this study. Furthermore the result of this study can be used as a benchmark for future experiments and/or more detailed simulations such as all-atom MD simulation. In all the cases considered in this study, a membrane local curvature of *C*_*L*_ = 0.04 nm^-1^ was applied on the bilayer in both of bilayer principal directions (spherical bending). This value corresponds to a radius of curvature of *R = 1/C*_*L*_ = 25 nm, which is in the range of bilayer physiological curvatures reported in the literature [6, 31]. We used ABAQUS commercial software (Abaqus/Standard; Simulia Dassault Systemes Simulia Corp. Providence, RI, USA) for our FE computations.

### Free energy calculations of MscL gating

Herein we consider a rudimentary free energy equation, where only the contributions of the free energy term related to hydrophobic mismatch and local bending are present.

$$G=G_{H}+\;G_{B}$$ (1)

With *G*_*H*_ and *G*_*B*_ are the general free energy associated with the hydrophobic mismatch and local bending contributions, respectively. Other possible free energy terms such as the contributions of external membrane tension, intrinsic local curvature and lipid-protein phase mixing do not appear in our final equation. This is because there is no external tension applied on the bilayer in our simulations. Also, in our system only a single protein is assumed to be reconstituted in an initially flat bilayer. Therefore, the contribution of the intrinsic local curvature of the lipid and the entropy term related to clustering and mixing of MscL with lipid molecules are negligible too.

Consequently, combining the relatively simple but efficient functions for the free energy contributions of hydrophobic mismatch [39] and local bending of the membrane and the protein [40], Eq 1 can be expressed as Eq 2. This equation can be used as an alternative mean-field approach to study the effect of lipid bilayer on not only MS channels but also on other membrane inclusions.

$$G=\left[\sqrt{2}\;{\left(\frac{K_{A}^{3}K_{B}}{t^{6}}\right)}^{\frac{1}{4}}{\left|\frac{d_{p}-d_{l}}{2}\right|}^{2}.2\pi R_{ave}\right]+\left[\frac{K}{2}{\left(C_{L}-C_{P}\right)}^{2}+K^{\prime }\left(C_{L}^{*}{}^{2}-2C_{L}^{*}C_{P}^{*}\text{cos}\left(2\theta \right)+C_{P}^{*}{}^{2}\right)\right]$$ (2)

Where *K*_*A*_ and *K*_*B*_ are the areal expansion and bending moduli of the lipid bilayer. *d*_*p*_ and *d*_*l*_ are the hydrophobic lengths of the protein and the bilayer, respectively. *t* is the thickness of each monolayer and *R*_*ave*_ is the average radius of the channels. *K* and *K'* are two independent coefficients related to the protein-lipid interaction. *θ* is the angle between the intrinsic principal axis of the protein curvature and that of the membrane curvature, which for MscL and the lipid bilayer (POPC) can be assumed to be zero. *C*_*L*_ *= (C*_1_ *+ C*_2_)/2 is the mean of the bilayer principal curvatures *C*_1_ and *C*_2_. $C_{L}^{*}=(C_{1}-C_{2})/2$ is the difference between the two curvatures, which is zero in the case of spherical membrane bending. Note that in this study by bilayer curvature we mean the curvature of the bilayer midplane. We have similar definitions for the protein principal curvatures as well, *C*_*P*_ = (*C*_*P*1_ + *C*_P2_)/2 and $C_{P}^{*}=(C_{P1}-C_{P2})/2$. Moreover, some assumptions can be made to further simplify Eq 2. For instance, a ratio of $\frac{K^{\prime }}{K}=1$ can be assumed, as it has been used for KvAP channel in the bilayers [41]. Also we induced spherical curvature, *C*_1_ = *C*_2_ = *C*_*L*_. Moreover, based on the continuum shape of MscL in the closed and open states, MscL is treated as an isotropic inclusion, i.e. its shape is either cylindrical or conical in both principal directions with the same radius of curvature. Thus we can assume *C*_*P*1_ = *C*_*P*2_ = *C*_*P*_.

## Results

**Fig 2:**
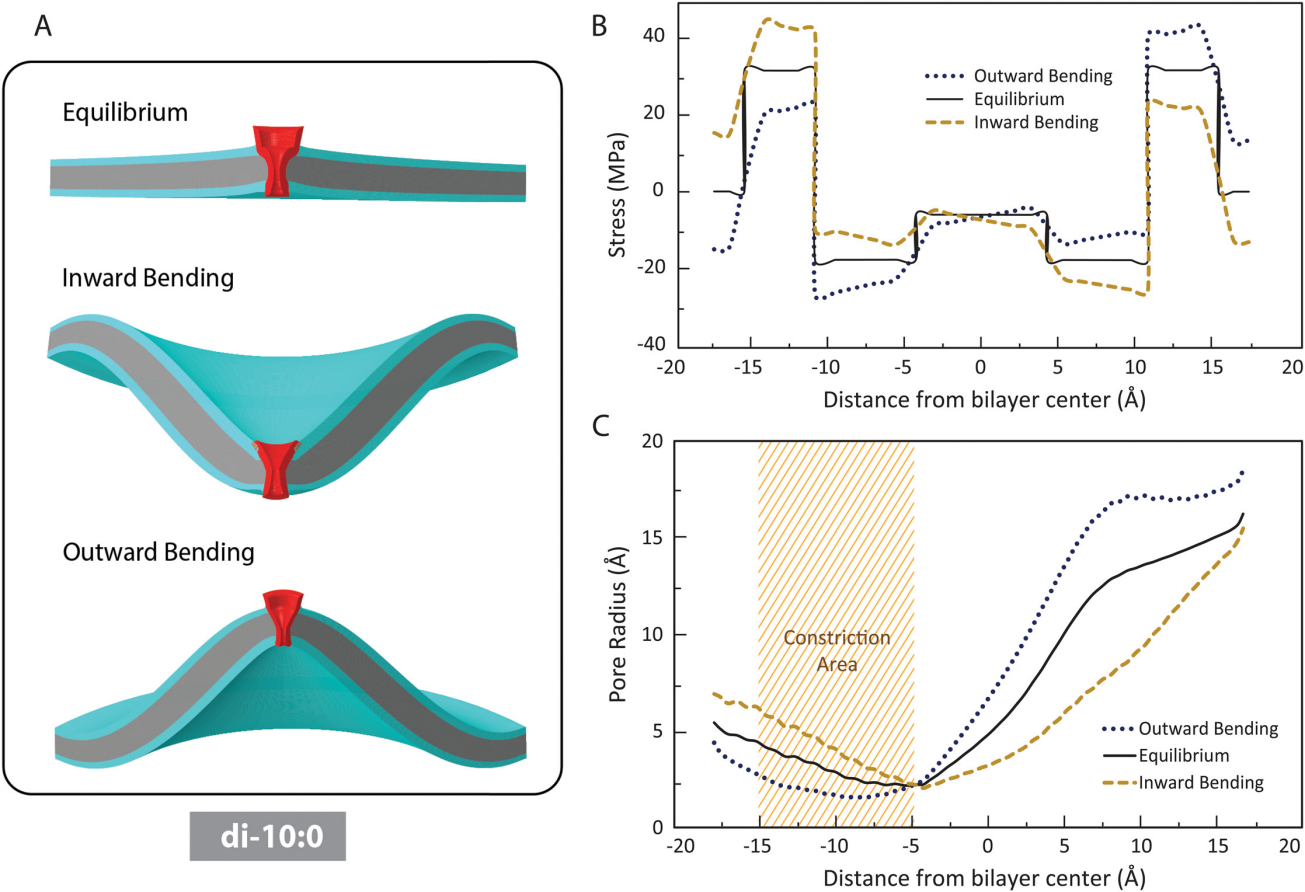
The influence of inward (negative) and outward (positive) membrane bending on the pressure profile of di-10:0 bilayer (positive mismatch) and the MscL pore shape. (A) Lipid bilayer deformation due to the intrinsic bilayer pressure profile (top), inward local bending (middle) outward local bending (bottom) of the membrane. (B) Inward local bending causes an asymmetry in the pressure profile such that the total tension in the inner monolayer is higher than tension in the outer monolayer. In contrast, outward bending causes more tension in the outer monolayer. (C) Compared to the equilibrium shape, inward bending results in a considerable opening in the narrowest part of the channel (shaded region which structurally encompasses L19 and V23), whereas the outward bending narrows the channel pore in this area.

**Fig 3:**
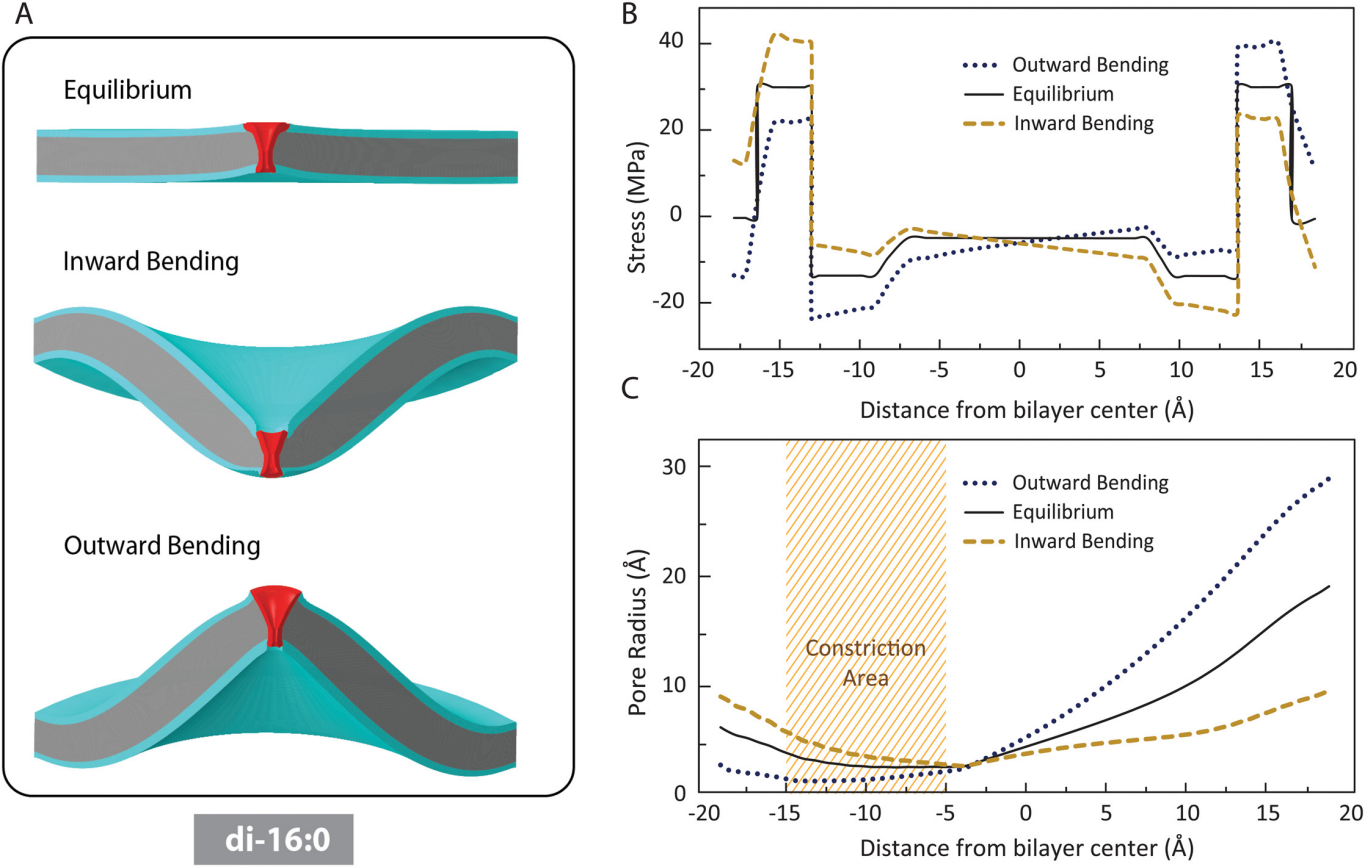
The effect of inward and outward bending on the channel conformation when there is no protein-lipid mismatch. (A) Membrane (POPC di-16:0) deformation after the MscL reconstitution (top panel), after an inward local bending (middle panel) and after an outward local bending (bottom panel). (B) Asymmetry in the pressure profile due to local bending. As a result of inward local bending, the total tension generated in the inner leaflet is higher than the membrane tension in the outer one. In the contrary, outward bending causes more tension in the outer monolayer compared to the inner one. (C) Compared to the equilibrium shape, inward bending results in a considerable opening in the narrowest part of the channel (shaded region which structurally encompasses the hydrophobic pore), whereas the outward bending inhibit the channel in this area.

**Fig 4:**
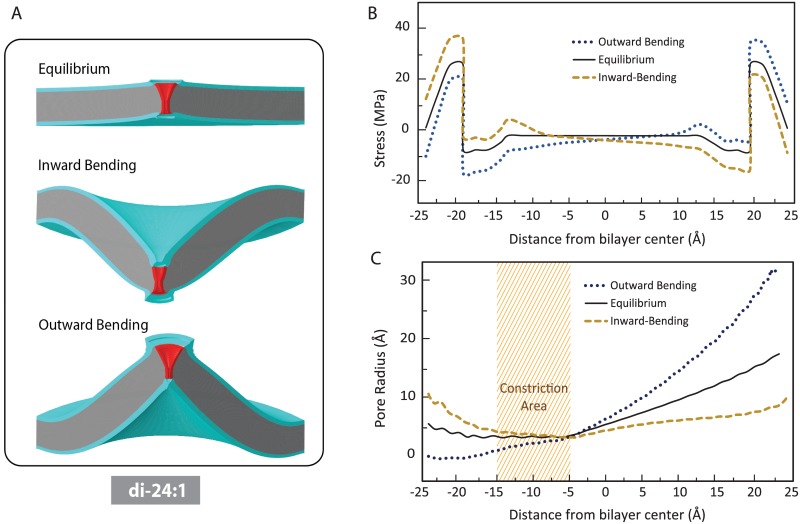
The influence of inward and outward local bending on the pressure profile of POPC di-24:1 bilayer and the MscL pore shape (negative hydrophobic mismatch). MscL is equilibrated due to the intrinsic pressure profile of the lipid bilayer. (A) Lipid bilayer deformation due to intrinsic pressure profile of lipid bilayer (top panel), inward bending (middle panel) outward bending (bottom panel). (B) The peak stress in the pressure profile is smaller in the thicker bilayers. Similar to the previous cases, inward local bending causes an asymmetry in the pressure profile such that the total tension in the inner monolayer is higher than in the outer monolayer. In contrast, outward local bending causes more tension in the outer monolayer. (C) Based on the channel pore in the equilibrium state, inward bending results in expansion of the narrowest part of the channel (shaded area), whereas the outward bending narrows the channel in this area.

Our FE simulations examined the effect of positive and negative local membrane bending on the MscL pore shape in three different cases; (i) positive protein-lipid hydrophobic mismatch (i.e. thinner lipid) (Fig 2); (ii) zero mismatch (Fig 3); and (iii) negative hydrophobic mismatch (i.e. thicker lipid) (Fig 4).

MscL is firstly deformed to equilibrate with the intrinsic lateral pressure profile of a thin membrane (e.g. di-10:0 POPC bilayer) (Fig 2A). Consequently, the MscL pore shrank in the hydrophobic constriction region (narrowest part of the pore) compared to its initial shape in the detergent (taken from the shape of the crystal structure). At the molecular level, the pressure in this area is caused by the steric repulsion of the lipid tails.

However, the pore is slightly widened in the regions away from the core due to the pressure regime in the head group region. Since MscL’s hydrophobic length in the case of positive mismatch is longer than the lipid hydrophobic thickness, the external boundaries of the protein do not deform considerably. The membrane around the channel area slightly curves upwards as a result of MscL insertion into the membrane, consistent with the previous studies [6].

More importantly, we observed that inward (negative) local bending of the membrane caused an asymmetry in the bilayer pressure profile such that the total tension in the inner monolayer, $\gamma =\int_{-t}^{0}P\left(z\right)dz$, was larger than in the outer monolayer (Fig 2B). Where, *P(z)* is the lateral pressure profile of the bilayer. In contrast, outward (positive) membrane bending caused a higher tension in the outer monolayer compared to the inner layer. Conceptually, this is a bilateral cause-and-effect meaning that if one asymmetrically changes the pressure profile in one layer versus another (e.g. asymmetric incorporation of amphipaths), the bilayer curves in response to the tension imbalance between the monolayers.

Taking into account the geometry of the MscL pore in the resting state, inward bending resulted in a considerable opening in the narrowest region of the channel (the hydrophobic lock), whereas the outward bending narrowed the channel in this area (Fig 2A and 2C).

The equilibrated MscL pore under zero mismatch conditions (e.g. di-16:0 POPC bilayer) is shown in Fig 3A. As expected [42, 43], due to the pressure regime in the tail region of the bilayer, the MscL pore is narrowed in this region but it is extended away from the core where it experiences the pressure regime of the hydrophobic-hydrophilic interface. Also, the equilibrated MscL redistributes the lateral pressure profile of the membrane and thus varies the membrane shape. As a result of MscL insertion, the bilayer marginally curved upwards close to lipid-channel interface (Fig 3A). The effect of inward and outward bending on lateral pressure profile of the lipid under conditions of zero mismatch are shown in Fig 3B. Similar to what we observed with positive mismatch, inward local bending caused an asymmetry in the pressure profile such that the total tension in the inner leaflet was higher than in the outer leaflet. In contrast, outward bending caused more membrane tension in the outer monolayer compared to the inner one. Furthermore, once again inward bending seemed more favorable for MscL gating than outward bending (Fig 3C).

Using our FE results, we can obtain the areal difference between the outer leaflet of the bilayer versus the area of the inner monolayer. To cause a curvature as shown in Fig 3A, the areal difference between the monolayers obtained from FE simulation is ~ 511 Å^2^. Having the values of the surface area of LPC and POPC, the number and molar percentage of LPC that can produce reported local bending in this study can be estimated. Assuming the area projection of the polar headgroup of LPC in a POPC bilayer to be 101.35 Å^2^ [44], to create a change in the area of one of the bilayer leaflets such that it causes a curvature of 0.04 nm^-1^ (Fig 2), 5 LPC molecules equivalent to a molar ratio (LPC/POPC) of 3.4% is required.

The effect of inward and outward local bending on the pressure profile of a thick lipid bilayer (POPC di-24:1) and the MscL pore shape (negative hydrophobic mismatch) is shown in Fig 4. The MscL pore is equilibrated due to the intrinsic pressure profile of the lipid (Fig 4B and 4C). However expectedly, the change in the protein conformation in the equilibrium (resting) state is less than in the other two cases (positive and zero mismatches) (Fig 4C). This is mainly due to the fact that the peaks in the pressure profile of the thickest lipid bilayer are smallest compared to the other lipids (Fig 4B and [45]). Similar to the previous cases, inward bending resulted in a considerable opening in the narrowest part of the channel, whereas outward bending narrowed the channel in this area and thus it inhibits channel gating.

**Fig 5:**
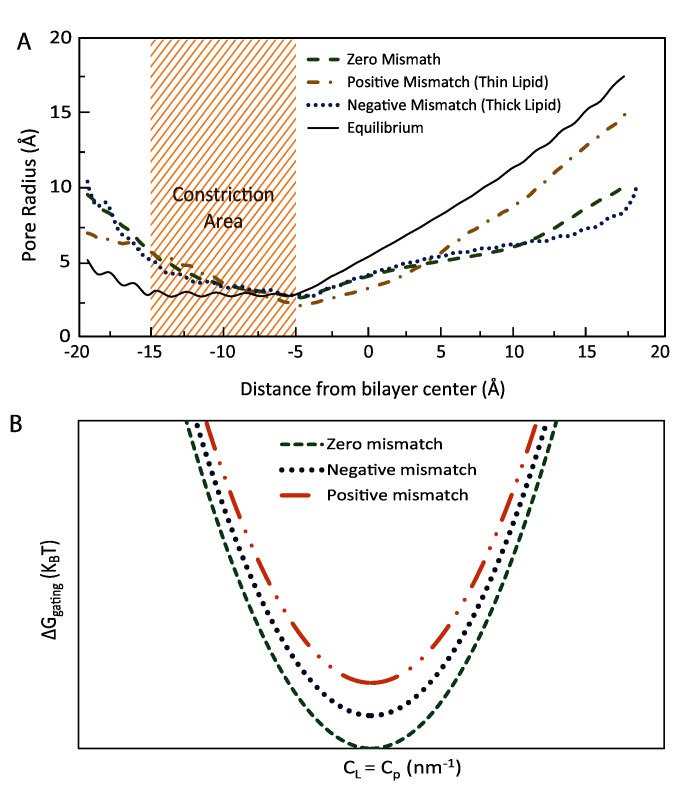
Computational and theoretical comparison of the effect of inward local bending on the channel pore domain for three different protein-lipid mismatches. Three possible protein-lipid hydrophobic mismatches can be positive, zero and negative. (A) A comparison has been made between the pore shapes in different lipid protein hydrophobic mismatches for inward local bending of the membrane. Overall, MscL in the bilayer with the same hydrophobic length shows a more expanded channel pore (shaded area) compared to positive and negative hydrophobic mismatches. (B) The gating free energy versus membrane has been shown for the above mentioned three possible lipid-protein hydrophobic mismatches. For a specific radius of curvature, the gating free energy is lower for zero hydrophobic mismatch compared to the other two cases. In turn, negative mismatch (thick lipid) results in lower free energy levels required for the MscL gating compared to the positive mismatch (thin lipid).

A comparison has been made between the pore shapes in different lipid protein hydrophobic mismatch for inward bending. Overall, MscL in the bilayer with the same hydrophobic length (zero mismatch) shows a more expanded state compared to positive and negative hydrophobic mismatches (Fig 5A). Moreover, MscL in thicker lipids (negative mismatch), is more expanded compared to MscL in the positive mismatch condition.

Finally, we examined all the different cases studied by FE simulation, using the energetic Eq 2, to see if the free energy of gating matches our computational observations. If one uses the available values for typical lipid bilayers in the literature, the free energy cost between the open and closed state of MscL with respect to curvature, for different lipid thicknesses can be illustrated as in Fig 5B. As can be seen, when there is no membrane curvature (*C*_*L*_*-C*_*P*_ = 0), the free energy cost for all three cases is minimal. More interestingly, the gating free energy for conditions of zero mismatch have a lower level than when there is a protein-lipid hydrophobic mismatch. This is mainly due to the additional free energy cost which will be added to the overall energy cost in the case of a hydrophobic mismatch between the inclusion (protein) and the bilayer (see Eq 2). In addition, the gating free energy cost for a negative mismatch is lower than a positive mismatch. This has a very interesting energetic consequence. It has previously been shown that the areal elasticity of the bilayer, *K*_*A*_, changes negligibly with thickness [46]. However, the bending rigidity of the membrane is proportional to the third or second order of bilayer thickness [22, 46]. Therefore, from the Eq 2, it can easily be inferred that the gating free energy level is lower for thicker bilayers compared to the thinner ones. This can be interpreted from the gating strain energy point of view as well. As the flexural rigidity is proportional to the third or second order of the bilayer thickness, a larger work (strain energy), $W=\frac{K_{B}A_{0}}{2R^{2}}$, is done to curve the membrane compared to what is required for thinner bilayers. Hence, it has a larger effect on the channel. Here *W* is the spent work to bend the bilayer with the initial area of *A*_*0*_ and *R* is the target radius of curvature [47], which is 25 nm in this study. Note that this is only important when there is a non-zero hydrophobic mismatch. Therefore, for a specific radius of curvature, a negative mismatch (thick lipid) results in lower free energy levels required for the MscL gating (Fig 5B).

## Discussion

In addition to physiological processes such as cell division, endocytosis and exocytosis, asymmetric incorporation of amphipaths into the lipid bilayer causes severe local membrane bending as well as membrane thickness alteration. Local curvature has been suggested experimentally to modulate numerous mechanosensitive (MS) ion channels by changing the pressure profile of the bilayer [10, 17, 18, 24]. Also it has been shown that MS channels such as MscL are more sensitive to membrane tension in thinner membranes due to hydrophobic mismatching concept [17, 22]. However, the dual effect of both of these physical factors, membrane curvature and hydrophobic mismatch, on gating of MS channels has not been investigated previously. Here using an FE framework we indicate that local bending of the membrane in a protein-lipid bilayer system with zero hydrophobic mismatch is more favourable for MscL gating compared to when there is a hydrophobic mismatch in the system. Moreover, in agreement with our previous results [5], the energy cost in both bending directions are similar, yet we observed that inward bending (negative bending) is more effective for MscL opening compared to outward (positive) bending. Intriguingly, negative mismatch conditions resulted in a more expanded pore compared to a positive mismatch condition. Previous experimental results obtained by EPR spectroscopy also demonstrate that the maximal negative hydrophobic mismatch in PC10:1 bilayers was insufficient to open MscL [17]. Although the outcomes of both studies are similar, the mechanism of pure tension [17] is likely to be different from that of local bending (examined in the current study). In pure membrane tension conditions, both monolayers are stretched (although one leaflet is less stretched than the other depending on the patch configuration [22]). However, in the case of local curvature, one monolayer can be stretched while the other one is compressed depending on the curvature direction. Hence, this causes the membrane to fold upward (positive) or downward (negative) as a response to the asymmetry of the force between the monolayers [48]. As shown in Fig 1B, for example in inward bending, the lower leaflet is stretched, whereas the upper leaflet is under compression.

Using a lysolipid like LPC as our additive model, our FE modelling data and the theoretical model provided by Markin & Martinac1991[49], we were able to estimate quantitative values for the amount of LPC required to be added to a leaflet of a lipid like PC:16 (zero mismatch) to cause a local curvature that can modulate MscL activity. For example based on our FE results, we need ~ 3.4% molar of LPC to be added to the inner leaflet of a PC16 bilayer to expect a negative (inward) curvature of 0.04 (nm^-1^) and thus having an effect on the channel pore as shown in Fig 3. This is lower but in the range of experimentally used LPC for MscL activation [17, 27].

It would be interesting to verify our computational outcomes in future studies such that, for example a conical shape molecule like LPC may be asymmetrically inserted into lipid bilayers with different chain lengths (thicknesses) and look at the MscL activity changes in these systems.

Finally, we confirmed our results by providing an energetic equation that accounts for the gating free energy as a function of local membrane bending and protein-lipid hydrophobic mismatch. Given the importance of these physical stimuli in physiological settings it is essential for our understanding of mechanosensitive channels to determine quantitatively how they converge and modulate channel function. Future studies such as this using MscL as a model MS channel may also provide insight into the function of other prokaryotic and eukaryotic membrane proteins.
